# Supplementary material for: How do we tread? Differences in stability-related foot placement control between overground and treadmill walking in young adults
Source: PLoS One. 2026 Mar 24;21(3):e0344704. doi: 10.1371/journal.pone.0344704 (PMC13012486; doi:10.1371/journal.pone.0344704)
Supplement: S1 File — (PDF) [file pone.0344704.s001.pdf]

S1

Variability of CoM position and velocity

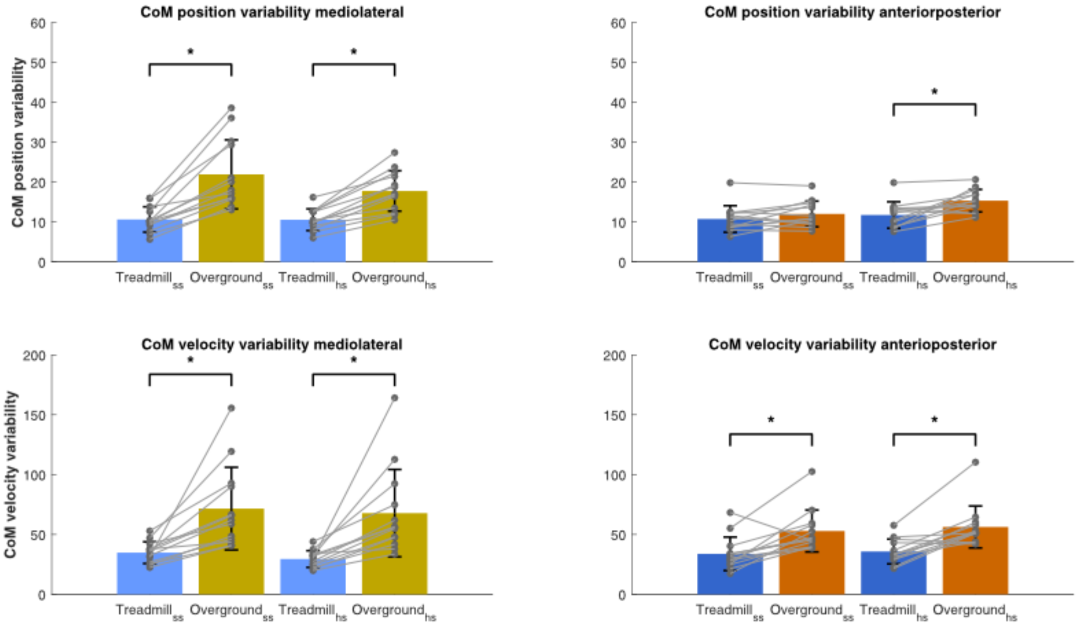

S1 Fig 1. Variability of CoM$_{pos}$ and CoM$_{vel}$ in mediolateral and anteroposterior direction, comparing treadmill and overground walking. *Mean variability is depicted for at the start of the step and at heel strike. Error bars represent the standard deviation and grey dots the individual data points. *$p < 0.05$.*
