# Supplementary material for: How do we tread? Differences in stability-related foot placement control between overground and treadmill walking in young adults
Source: PLoS One. 2026 Mar 24;21(3):e0344704. doi: 10.1371/journal.pone.0344704 (PMC13012486; doi:10.1371/journal.pone.0344704)
Supplement: S2 File — (PDF) [file pone.0344704.s002.pdf]

S2

Foot placement error (n-2 steps before turn)

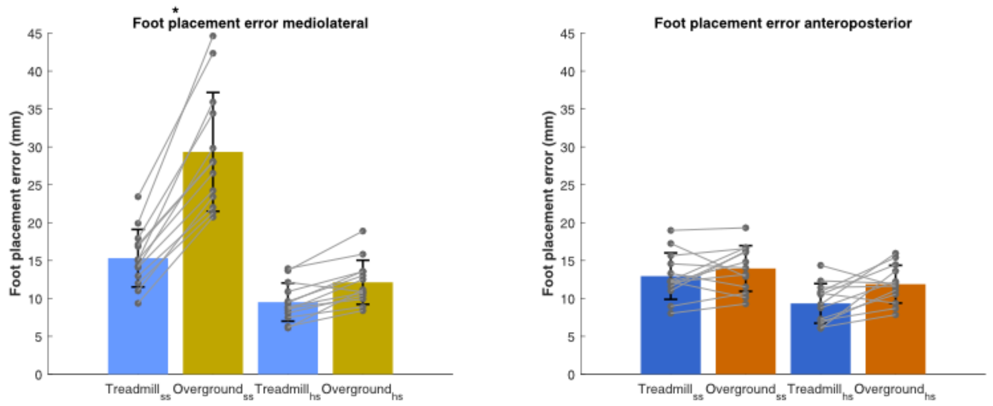

S2 Fig 1. Comparison of ML and AP foot placement errors between treadmill and overground walking with n-2 steps removed before turns. *Foot placement errors (i.e. the standard deviation of the residuals in millimeters), as a measure for foot placement precision are depicted for the predictions at the start of the step and at heel strike. Error bars represent the standard deviation and grey dots the individual data points. *p < 0.05.*
