# Supplementary material for: How do we tread? Differences in stability-related foot placement control between overground and treadmill walking in young adults
Source: PLoS One. 2026 Mar 24;21(3):e0344704. doi: 10.1371/journal.pone.0344704 (PMC13012486; doi:10.1371/journal.pone.0344704)
Supplement: S3 File — (PDF) [file pone.0344704.s003.pdf]

S3

Progression of foot placement errors over trial

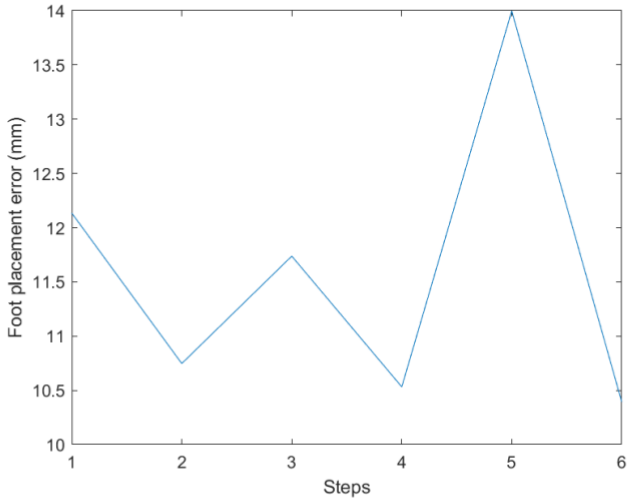

S3 Fig 1. Example of progression of foot placement errors over the trial (10m walking path) in mediolateral direction. *Mean foot placement errors at heel strike are depicted for each step averaged over all trials of one participant.*
