# Supplementary material for: How do we tread? Differences in stability-related foot placement control between overground and treadmill walking in young adults
Source: PLoS One. 2026 Mar 24;21(3):e0344704. doi: 10.1371/journal.pone.0344704 (PMC13012486; doi:10.1371/journal.pone.0344704)
Supplement: Attachment — Submitted filename: Response to Reviewers.docx [file pone.0344704.s005.docx]

**Reviewer #1:**

The Authors would like to thank the Reviewer for their feedback to our manuscript. A brief point-by-point response to Reviewers’ comments is presented below. To ensure clarity throughout, the Reviewers’ comments are presented in blue, with our responses in an indented format and a black font. All changes made to the manuscript are presented under quotes in black font and highlighted in yellow and the corresponding page and line numbers of the revised manuscript are denoted in red and bold.

This study investigates the differences in foot placement control mechanisms between treadmill and overground walking in young healthy adults (N=13). Utilizing linear models that predict step width and length based on Center of Mass (CoM) state, the authors tested hypotheses regarding the existence, precision, and strength of these control mechanisms across conditions.
The results suggest that while foot placement control is active in both conditions, it manifests differently: overground walking is characterized by lower precision (larger residuals), wider steps, and a shift in reliance from CoM position to CoM velocity feedback. The authors conclude that caution is required when generalizing treadmill findings to real-world gait.

General Assessment
This is a well-written and logically structured manuscript that addresses a significant gap in the biomechanics literature: the ecological validity of treadmill-based stability research. The methodology regarding the foot placement model is grounded in established literature, and the statistical approach (using Linear Mixed Models to control for speed/step width confounds) is robust.
However, there are valid concerns regarding the specific experimental constraints of the overground condition (the Figure-8 path) and the sample size that need to be addressed to ensure the validity of the conclusions.

Major Comments
1. The Steady-State Assumption in a Figure-8 Path
The overground condition involved walking in a Figure-8 path, with analysis restricted to the straight segments. The authors removed the last step of the straight path to minimize anticipation effects. Though is is known that anticipatory locomotor adjustments for turning often begin several steps prior to the turn itself (e.g., changes in trunk roll, step width modulation). In a 10-meter straight section, excluding only the last step may not be sufficient to capture true "steady-state" walking comparable to the treadmill condition. Can the authors provide data or citations demonstrating that the steps analyzed (steps n-2, n-3, etc.) do not show preparatory kinematic changes (such as CoM deviation toward the turn direction). If this cannot be demonstrated ok,but i think this limitation should be discussed more prominently as a potential confounder for the increased "error" (residuals) found overground.

We appreciate the reviewer’s comment. Several steps in our post-processing were implemented to minimize potential effects related to gait transitions before and after turns. Specifically, the turns in the figure-8 walking task are relatively large around 3 cones placed in a triangle and occur outside the calibrated capture volume of the motion-capture system. During data collection, investigators manually initiated and stopped each straight-walking trial, pressing “start” only when the participant entered the capture volume with all relevant markers visible and “stop” once the markers began to leave the field of view. During labelling, only straight-line walking sequences were retained, and all turning steps were trimmed from the data. In a final processing step, one step before and one step after each turn were additionally removed to further minimize any potential residual effects of turning. We further repeated the analysis of foot placement errors during overground walking with an additional step removed before each turn (Figure 1) and the result was unchanged compared to our results presented in the manuscript with just one step removed. Based on this and the multi-stage procedure during processing, we are confident that the analysed data represent steady-state walking unaffected by turning transitions. Nonetheless, we acknowledge that this approach may not fully eliminate the residual effects of turning, as these were not systematically analysed in the present study, and we extended this point in the Limitations section.

“Therefore, some of the steps included might not reflect steady-state walking, potentially contributing to larger residual errors in overground walking. However, when repeating the analysis of foot placement errors during overground walking with an additional step removed before each turn, the result was unchanged compared to our results presented with just one step removed (S1 Fig 2).” (**p. 23, lines 550-554**)

Figure 1: Comparison of ML and AP foot placement errors between treadmill and overground walking with n-2 steps removed before turns.

2. Interpretations of Velocity Gain βvel
The study reports a higher relative contribution of velocity feedback during overground walking. On a treadmill, the belt speed is fixed. While the subject can fluctuate relative to the belt, the global velocity is highly constrained. Overground, the subject has degrees of freedom to modulate speed freely. I believe that it is crucial to distinguish whether the increased βvel overground is a result of better sensory integration (as argued in the Discussion) or simply a mathematical result of there being more variance in velocity to regress against during overground walking. If the range of CoM velocity on the treadmill is negligible, the model may naturally suppress the βvel term.
Can the authos please provide descriptive statistics comparing the variance (or standard deviation) of the CoM velocity between the two conditions. If the variance is significantly different, the discussion should reflect that the "increased reliance" might be a function of the task constraints allowing velocity modulation, rather than purely a sensory re-weighting.

We tested the variance of the CoM velocity between overground and treadmill walking and confirm that there is a significantly higher variability in CoM velocity (Figure 2). However, we also observed an equally increased variability of CoM position during overground walking. Therefore, the higher relative contribution of velocity feedback than position feedback during overground walking cannot only be a mathematical result of more variance available.

Figure 2: Variability of CoMpos and CoMvel in mediolateral and anteroposterior direction, comparing treadmill and overground walking

We have adapted the paragraph, clarifying the two possible explanations (increased variance and sensory re-weighting).

“The significantly lower variability of CoM velocity observed during treadmill walking may therefore limit the velocity-related feedback available for foot placement adjustments due to task constraints.” (**p.21, lines 486-488**)

and

“Our results support the hypothesis that foot placement control differs between treadmill and overground walking as can be seen by changes in CoM feedback contribution. Although an increase in variance could mathematically inflate the contribution of CoM velocity, the variance of CoM position was increased as well (S1 Fig 1). The former can therefore not fully explain the changes in partial correlations between conditions, making a sensory re-weighting more likely.” (**p.21, lines 491-496**)

Furthermore, we have added the graph to the supplementary material.

3. Sample Size and Power
The final analysis included 13 participants. While the authors state that the number of steps (n=130) is sufficient for the model to plateau, the statistical power to detect differences between conditions (treadmill vs. overground) relies on the number of participants. Given the high inter-subject variability often seen in gait strategies, N=13 is on the lower end for a study generalizing to a broad population. If a power analysis was conducted a priori, please report it. If not, acknowledge the small sample size as a limitation specifically regarding the generalizability of the LMM results, particularly for the AP direction where results were more variable.

We thank the reviewer for raising this important limitation of our study. No formal a priori sample size calculation was performed for the original study. As the present work represents a secondary analysis of this existing dataset, no additional sample size calculation was performed. We agree that the small sample size is a limitation of our study and limits its generalizability, and acknowledge this in the limitations section.

“The sample size of 13 participants is relatively small, but the number of steps included is well above the value for which foot placement outcomes tend to plateau (23). Nevertheless, the generalizability of our results to a broader population may be limited, especially considering the high variability of results in the AP direction.” (**p.23, lines 542-545**)

4. Coordinate System Rotation and Drift
The authors note that marker trajectories were rotated to align with the walking path, but "a slight drift remains visible". In a linear regression model where CoM is a predictor, systematic drift could artificially inflate the residuals or skew the position gain
Please clarify if the data were de-trended beyond the simple rotation. If the drift correlates with the progression along the 10m path, it acts as a structured noise. A sensitivity analysis (e.g., checking if residuals increase linearly over the trial) would strengthen the validity of the "lower precision" finding.

We did a visual check of the residuals over the trials, and no linear increase was visible. We therefore decided to refrain from a more detailed analysis. However, we have added this observation in the limitations section and the graph to the supplementary material.

“Another concern might be that, even after rotating the marker trajectories, a slight drift remains visible (Fig 1), which could potentially affect the results of the foot placement model. However, foot placement errors did not increase linearly over the trial (S1 Fig 3).” (**p.23, lines 554-557**)

Figure 3: Example of progression of foot placement errors over the trial (10m walking path) in mediolateral direction. Mean foot placement errors at heel strike are depicted for each step averaged over all trials of one participant.

Minor Comments
1. Explanation of i=1 vs i=51:
The distinction between "feedback control" (at i=1, start of swing) and "performance" (at i=51, foot placement) is mentioned. However, for readers less familiar with this specific modeling approach, it would be helpful to explicitly state why the start of the swing phase represents feedback planning, whereas the end represents the execution/outcome.

We have added the following explanation to the methods:

“Measures derived from the model fitted for i=1 (i.e. with the CoM state at the start of the step as predictors) can be considered as measures of feedback control (24), since at the start of the step the CoM state has been shown to be a better predictor of imminent foot placement than the state of the swing foot itself (11). This suggests early feedback control based on sensory information of the CoM state. Moreover, muscle activity during early swing drives step-by-step foot placement control (25, 26), providing further support of feedback control relying on early swing CoM state information. At heel strike (i=51), i.e. the instance of foot placement, the model captures the result of this feedback control. At the instance of foot placement, the coordination between the CoM and its base-of-support is realized (5, 11, 27). Therefore, measures derived from the model fitted for i=51 (i.e. with the CoM state at heel strike) can be considered as performance measures of stability-related foot placement control.” (**p. 9, lines 207-217**)

Recommendation
Major Revision.
The study offers valuable insights, but the interpretation of the results - specifically regarding the Figure-8 constraint and the mathematical influence of velocity variance – in my view needs to be more rigorously defended to support the conclusions.

We thank the reviewer and refer to the additional analysis and answers provided above.

**Reviewer #2:**

The Authors would like to thank the Reviewer for their feedback to our manuscript. A brief point-by-point response to Reviewers’ comments is presented below. To ensure clarity throughout, the Reviewers’ comments are presented in blue, with our responses in an indented format and a black font. All changes made to the manuscript are presented under quotes in black font and highlighted in yellow and the corresponding page and line numbers of the revised manuscript are denoted in red and bold.

The current manuscript tested the idea that step width and step length is modulated by the nervous system to ensure stability. Previous studies have primarily tested this idea on a treadmill while most walking by individuals is completed overground. Individuals walking overground and on a treadmill. A linear statistical model was created whereby step width was modeled as a function of center of mass position and velocity. Differences between the two walking conditions were detected specifically the authors report lower foot placement precision overground vs. treadmill with greater sensitivity in center of mass velocity compared to position. The topic is of interest to the general gait and biomechanics community. There are a few considerations with the manuscript in its current form that should be addressed.

1. Overall the introduction appears unnecessarily lengthy. While the information presented mostly relates to the purpose of the study, it could be tightened up for the reader. As an example paragraph 2 and 3 are very similar in topic and could be combined. Paragraph 4 and 5 are also similar and could be combined.

We reviewed paragraphs 2 and 3, and decided to retain most of the information and these separate paragraphs to retain clarity of some important concepts that keep coming back throughout the manuscript. However, we did attempt to make paragraph 3 less wordy by removing superfluous additions such as “…varying with task constraints” and “…further underscoring the need to examine it beyond treadmill walking.” (**p.4-5, line 79-98**)

In paragraph 4 we noticed that we were indeed repeating the purpose of our study a lot. To resolve this we removed superfluous parts such as “As a result, foot placement findings from treadmill studies may not directly explain the increased fall risk or impaired gait stability observed in daily life.” , “Investigating foot placement control during overground walking, and its potential differences from treadmill walking, is an important step toward understanding impaired gait stability and its underlying mechanisms in daily life contexts”, and “Motivated by these considerations, we investigated differences in foot placement strategies between overground and treadmill walking”. Moreover, we merged what remained of paragraph 4 with paragraph 3. (**p.4-5, line 79-98**)

In addition, we have removed most of paragraph 5 (as it mostly rephrased what had been discussed before) and merged some of the information of that paragraph with our second and third hypotheses, to further shorten the introduction.

2. The primary critique is that this work suggests that the nervous system is targeting a particular step width on a step-by-step basis and if that step is not achieved it must be an error, despite the fact that many step placements could still satisfy the criteria for stability. Under conditions in which constraints are provided where a specific step width or range of step widths are fixed (e.g., Rosenblatt et al. Exp Brain 2014, Sidaway Exp Ger 2025) it is sensible that the constraints imposed would require greater precision because error in foot placement negatively effects task completion. (Sawers & Ting G&P 2015). However, overground walking unconstrained has no such constraints. People can and do meander. Thus, is it possible that the argument for unconstrained walking places too much emphasis on precision?

It is true that even healthy adults make “foot placement errors” and that this is not a direct threat to their walking stability. However, that some imprecision can be allowed does not mean that “foot placement precision” in this context is not a measure of interest. In other work (Mahaki, van Leeuwen, Bruijn, van der Velde, & van Dieën, 2023), older adults showed a reduction in foot placement errors following a training intervention, alongside improved walking stability. Thus, the measure has shown to be sensitive enough to capture training effects. We refer to this work in the following sentence:

“These measures have shown to capture foot placement training effects during treadmill walking (15, 16).” (**p.4, line 74-75**)

In addition, to avoid creating an impression that every error is an immediate threat to stability we have changed the following sentence in our introduction from:

“…precise control mechanisms ensure a stable gait.”

To:

“…neural control mechanisms ensure a stable gait.” (**p. 3, line 49**)

Moreover, in the discussion we now mention:

“…we interpret the foot placement model to represent sensory feedback control, with the foot placement errors representing either motor noise or looser control...” (**p. 24, line 562-564**)

Lastly, the “foot placement precision” as discussed in the context of our manuscript (i.e. how precise participants adjust their foot placement with respect to deviations in their CoM state), should not be confused with “foot placement precision” defined as how precise participants are stepping on a stepping target. When having an additional constraint (e.g. a stepping target) stability-related foot placement control has shown to become less tight (e.g. Perry and Srinivasan (2017) and van Leeuwen, van Dieen, Daffertshofer, and Bruijn (2020)).

3. Introduction Line 47: The current study is not investigating falls or neurological diseases. The sample was 14 individuals with an average age of 24. Falls are less likely a concern. However, the manuscripts first paragraph describes falls and risk of falls. It is generally likely that whatever mechanism discovered here is likely not the same for people with high fall risk. Thus, the intro should focus on what we are learning about how the young nervous system controls stability. Consider moving patient population to the discussion.

In order to shift the focus towards the foot placement mechanism in healthy adults, we no longer open the introduction by mentioning patients. Instead we focus on what the mechanism does in healthy adults:

“…stability is maintained through effective coordination between the center-of-mass (CoM) and its base of support.” (**p.3, lines 47-48**)

We did retain the mentioning of populations in which this mechanism is impaired, to make the relevance of the study already clear from the introduction. However, we added a sentence to ensure the focus remains on healthy adults:

“To be able to put such impairments into context, foot placement control has been extensively studied in healthy adults (9-12), to firstly gain an understanding of the unimpaired mechanism.” (**p. 3. Lines 54-56**)

4. While walking speed on a treadmill maintains a consistent speed, overground walking provides no external constraints to the nervous system. During overground trials individuals were walking straight then turning then walking straight on a 4-minute loop. Was speed consistent for overground trials and if not, could that modify model estimates?

Changes in walking speed between trials of individual participants were within 3% of the mean value, for 3 participants within 5%, and are therefore considered consistent. Furthermore, no fatigue effects are expected for young, healthy adults within four minutes of walking and no visible changes in walking behavior were observed during the recordings. We have added the following lines to the limitations:

“While treadmill walking enforces a constant walking speed, walking speed during overground walking might vary between trials of a participant. However, as the participants were healthy young adults, fatigue-related effects on walking patterns were not expected, and intraindividual variations in walking speed were within 5% and therefore considered consistent.” (**p.23, lines 557-561**)

5. The use of the linear model is well reasoned and justified to predict foot placement, however the interpretation seems to suggest that whatever variance is not accounted for has a biological interpretation, i.e., error in foot placement. This seems like leap. This model included two terms to account for position and velocity of the center of mass. It may also be that this simple model does not account for other factors that could influence step-to-step adjustment. Please add this as a potential limitation of the model.

We have now added the following to the limitation section of the discussion:

“Another limitation that should be noted is that although we interpret the foot placement model to represent sensory feedback control, with the foot placement errors representing either motor noise or looser control, like any model, (part of) the residual might exist because the model does not fully capture the actual control parameters. The relatively high reported R2 of the model (9), suggests that the model is a meaningful representation of step-by-step foot placement control, even in this simple linear form. This is further supported by the sensitivity of the model in distinguishing foot placement control between different populations (3, 38) and following training interventions (15, 16). However, it cannot be dismissed that different or more complex models could potentially generate an even better representation. In its current form, the foot placement model generates residuals which can be interpreted to at least partly represent a foot placement error in the context of stability control. This is underscored by partial correction of this ”foot placement error” in healthy adults by subsequent center-of-pressure shifts during single stance through ankle moment control (39). When external stabilization alleviates the demands on stability control, execution of this corrective mechanism is diminished (40), underpinning that the residual of the foot placement model has implications for stability control.” (**p.24, lines 562-576**)

6. Terminal swing is typically considered in textbooks such the Neuman textbook (Kinesiology of the Musculoskeletal System) is considered 87-100% of the gait cycle. In the current manuscript the authors seem to consider this a specific instant and time where in the model terminal swing i=51. Is this 1 frame back prior to foot strike? This should be clarified for the reader.

Thank you for bringing this to our attention. Given the textbook definition we agree that “terminal swing” does not accurately describe i=51. Since our time-normalization covers one step from toe-off to heel strike and i=51 is the last sample, i=51 corresponds to heel strike. We have now replaced “terminal swing” by “heel strike” throughout the manuscript.

7. The discussion is very long and could benefit from tightening the language. Section 4.6 could be one or two paragraphs. As is it feels a bit bloated. Consider revising.

We have removed the following superfluous parts from section 4.6 of the discussion:

“A proper understanding of foot placement control during overground walking, and daily life gait, is essential for diagnosing and intervening stability control impairments.”

“The increased contribution of velocity feedback to foot placement control during overground walking is also highly relevant for assessing foot placement control in daily life.”

“Gaining insight into the differences in gait mechanisms between treadmill and overground walking is essential for explaining why gait outcomes often differ between laboratory and daily life conditions and for identifying potential reasons why treadmill-based interventions might not effectively improve gait stability in daily life.”

References:

Mahaki, M., van Leeuwen, A. M., Bruijn, S. M., van der Velde, N., & van Dieën, J. H. (2023). Mediolateral foot placement control can be trained: Older adults learn to walk more stable, when ankle moments are constrained. *PloS one, 18*(11), e0292449.

Perry, J. A., & Srinivasan, M. (2017). Walking with wider steps changes foot placement control, increases kinematic variability and does not improve linear stability. *Royal Society open science, 4*(9), 160627.

van Leeuwen, A. M., van Dieen, J. H., Daffertshofer, A., & Bruijn, S. M. (2020). Active foot placement control ensures stable gait: Effect of constraints on foot placement and ankle moments. *PloS one, 15*(12), e0242215.
